# 2-(1,2,4-triazole-5-yl)-1,3,4-oxadiazole as a novel building block for energetic materials

**DOI:** 10.3389/fchem.2022.996812

**Published:** 2022-08-26

**Authors:** Zheting Dong, Zhengqiang Wu, Qiang Zhang, Yuangang Xu, Guo-Ping Lu

**Affiliations:** ^1^ School of Chemistry and Chemical Engineering, Nanjing University of Science and Technology, Nanjing, China; ^2^ School of Chemistry and Life Sciences, Suzhou University of Science and Technology, Suzhou, China

**Keywords:** 1,2,4-triazole, 1,3,4-oxadiazole, energetic material, primary explosive, diazonium ylide

## Abstract

The exploration of novel nitrogen-rich heterocyclic building blocks is of importance in the field of energetic materials. A series of 2-(1,2,4-triazole-5-yl)-1,3,4-oxadiazole derivatives based on a new energetic skeleton have been first synthesized by a simple synthetic strategy. All three compounds are well-characterized by IR spectroscopy, NMR spectroscopy and thermal analysis. The compounds **5** and **8** are further characterized by single-crystal X-ray diffraction analysis. **8** and its salts (**8a**-**8c**) possess relative high decomposition temperature and low sensitivity, while **5** exhibits low decomposition temperature and high sensitivity. According to EXPLO5 calculation results of detonation performance, both **5** and **8** display acceptable detonation velocities (**D**) of 8450 m/s and 8130 m/s and detonation pressures (**P**) of 31.6 GPa and 29.2 GPa, respectively. Furthermore, **5** containing a rare diazonium ylide structure shows high impact sensitivity (4.5 J), making it has a potential as a primary explosive.

## Graphical Abstract

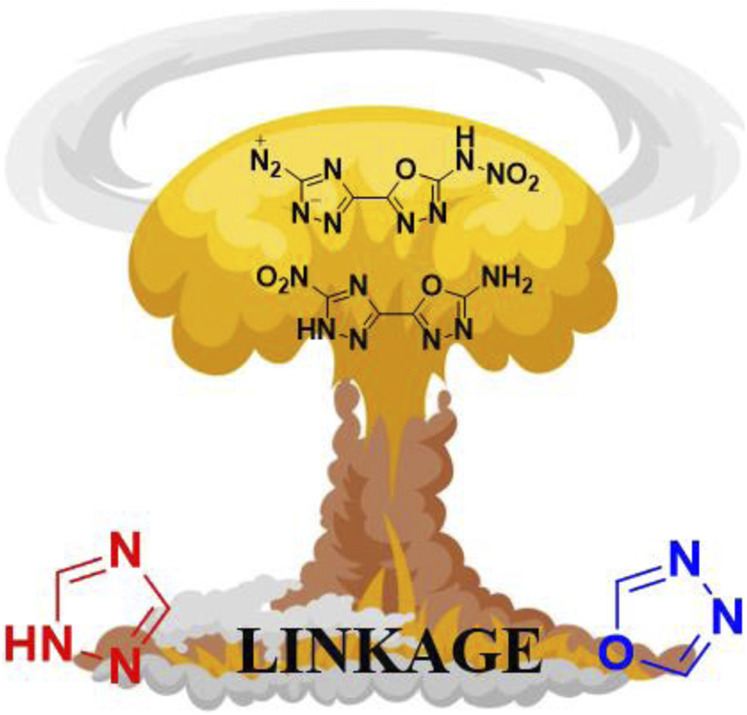

## 1 Introduction

Energetic materials are a class of special compounds that are extensively used in both military and civilian fields. In the past few decades, the development of new energetic materials has attracted considerable interest (Klapötke et al., 2007; Vo et al., 2014; Bélanger-Chabot et al., 2015; Zhang et al., 2017; Chen et al., 2018), and various high performance energetic materials have been disclosed. (Archibald et al., 1990; Zhang et al., 2000; Fischer et al., 2012) Although high detonation performance is always the primary requirement, there has been increased interest in the stability of energetic materials owing to the increase in the number of security-related issues in practical applications. (Klapötke and Witkowski, 2016; Tian et al., 2016) Thus, there is considerable demand to explore novel energetic materials that have a good balance between energetic performance and stability. (Xia et al., 2019) On the other hand, primary explosives and initiators represent a class of sensitive energetic materials that can be easily detonated by a small external stimulus such as flame, heat, impact, friction, electric spark, etc. (Deng et al., 2019) Lead azide (LA) and 2-diazo-4,6-dinitrophenol (DDNP) are the most widely used primary explosives, but both of them suffer limitations including high toxicity, much high sensitivity and poor detonation performance. (Giles, 2004; U.S. Environmental Protection Agency, 2014; Cao et al., 2016; Yuan et al., 2016; Wei et al., 2018)

Although the fabrication of energetic salts and crystals form known nitrogen-rich heterocyclic compounds are introduce to investigate next-generation energetic materials (Kumar et al., 2017; Tang et al., 2017; Yin et al., 2017), the construction of new covalent polynitrogen heterocyclic compounds is still the most fundamental area of this field. (Wang et al., 2019) In general, the combination of different N-heterocycles is one of the most significant methods to access novel energetic molecules. (Joo and Shreeve, 2010; Pang et al., 2018; Xu et al., 2018) 1,2,4-Triazole has been widely employed in the synthesis of energetic materials owing to its high enthalpy of formation. usually, amino group on 1,2,4-triazole is easier to convert into a diazo structure, resulting in a higher mechanical sensitivity of the whole system. The introduction of nitrogen-rich heterocycles into this framework is the common strategy to improve its stability. Meanwhile, the connection of nitrogen-rich heterocycles can increase the modifiability of energetic materials, so more explosive groups can be introduced into the energetic skeleton to improve their detonation performance. 1,3,4-oxadiazole has not received much attention in the field of energetic materials synthesis due to its low enthalpy of formation (51 kJ/mol). (Tang et al., 2015; Yu et al., 2017; Zheng et al., 2022) The formation enthalpy, detonation performance and stability of energetic materials can be effectively improved when 1,3,4-oxadiazole is connected with other nitrogen-rich heterocycles and explosive group (-NO_2_, -N_3_ and -NHNO_2_). In recent years, a large number of energetic materials with 1,3,4-oxadiazole rings linking other nitrogen-rich heterocycles and explosive groups with good detonation performance have been synthesized. (Zhang et al., 2017; Wang et al., 2019; Yan et al., 2020; Ma et al., 2021)

**SCHEME 2:**
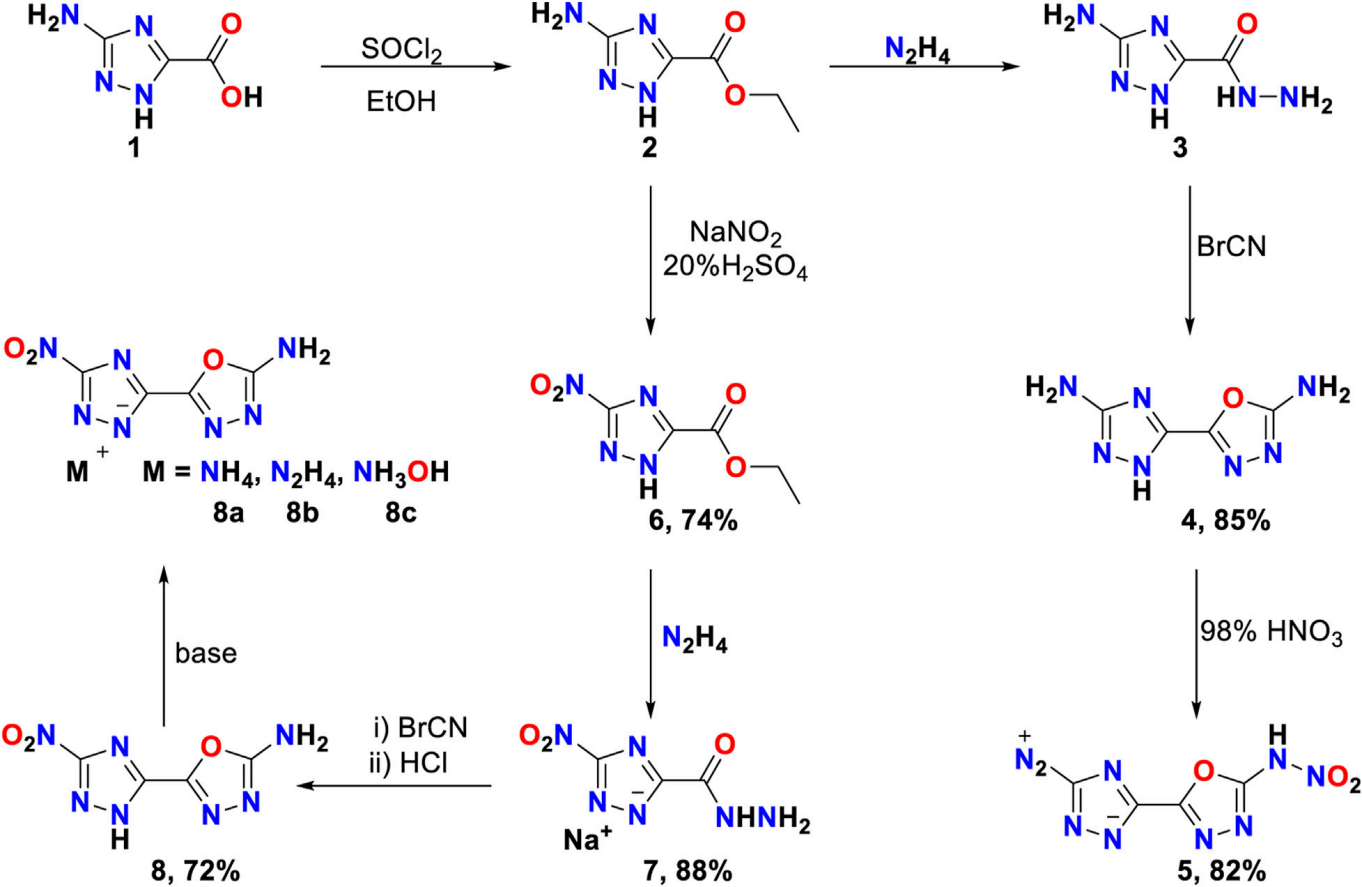
Synthesis of compounds 4–8 and 8a-8c.

With these above molecular properties as guidelines, we reason that the connection of 1,2,4-triazole and 1,3,4-oxadiazole can not only increase the formation enthalpy of energetic molecules, but also reduce their mechanical sensitivity. Herein, we have firstly disclosed the synthesis of 2-(1,2,4-triazol-5-yl)-1,3,4-oxadiazole derivatives by three-step reactions with high yields (Scheme 2). Interestingly, we accidentally obtained a diazonium ylide **5**, because HNO_2_ is generated during the nitration reaction, the amino group on the 1, 2, 4 triazole ring is more easily converted into a diazo group. Meanwhile, to further explore the nitration reaction conditions of compound 4, we tried different nitration conditions (Table 1). It might be a promising organic primacy explosive owing to its excellent detonation properties and certain sensitivity to external mechanical stimuli.

**TABLE 1:** Nitrification reaction conditions of 4

Nitrification reagent^a^	Time (h)	Temperature (°C)	Yiled (%)^b^
Fuming HNO_3_	72	25	-
Fuming HNO_3_/98% H_2_SO_4_	72	25	-
Fuming HNO_3_/TFAA	72	25	11
KNO_3_/98% H_2_SO_4_	72	25	0
98% HNO_3_	72	25	82

^a^ The ratio of mixed acid is HNO_3_ : H_2_SO_4_ = 1 : 1.^b^Products were determined by ^1^H,^13^C NMR, and single crystal X-ray diffraction. The yields of **5** was determined by LC.

## 2 Result and discussion

**SCHEME 1:**
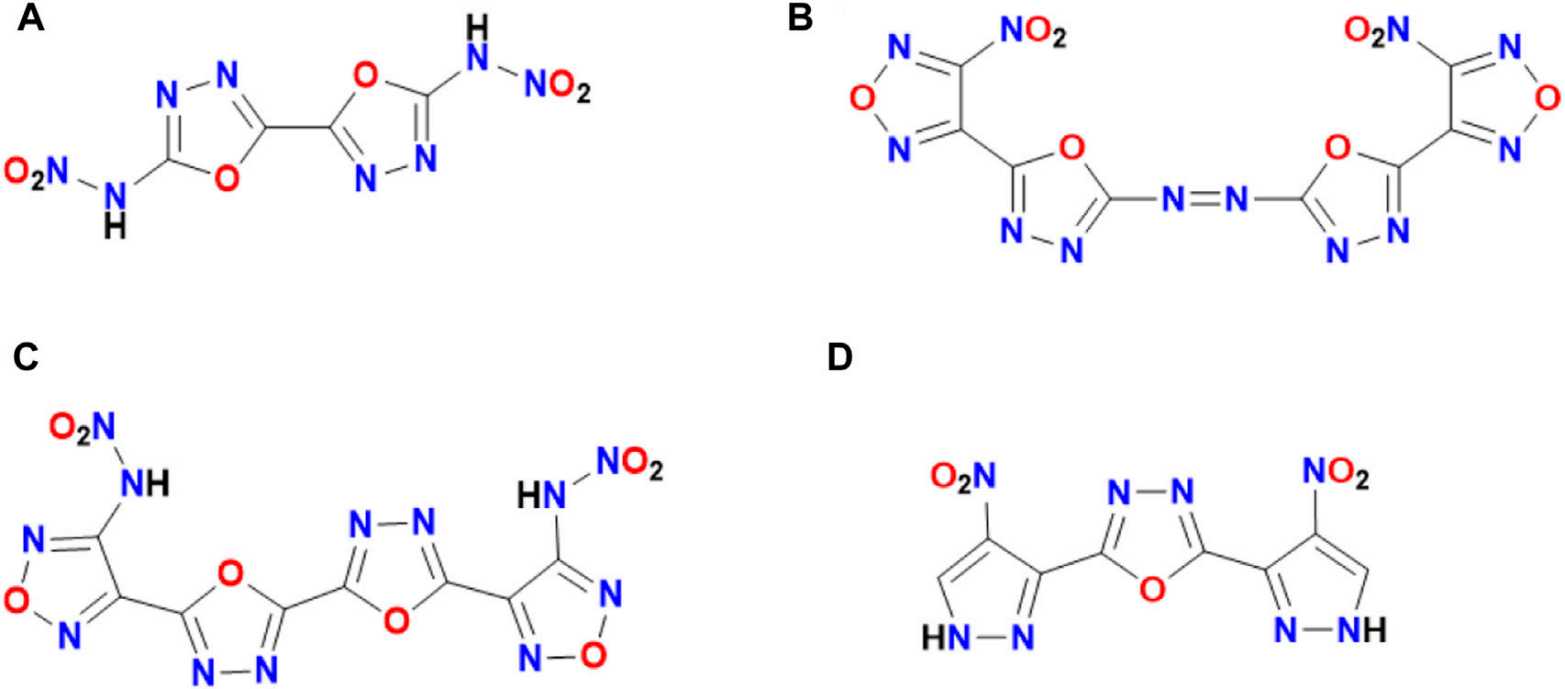
Energetic materials with 1,3,4-oxadiazole fragment.

The synthetic routes of compounds **5, 8, 8a, 8b** and **8c** are shown in Scheme 1. 5-Amino-1*H*-1,2,4-triazole-3-carbohydrazide **3** and sodium 5-nitro-1*H*-1,2,4-triazole-3-carbohydrazide **2** were prepared according to the previous method. (Dachs et al., 2013; Dippold and Klapötke, 2013; Zhang et al., 2018) Compounds **4** and **8** could be obtained by the reactions of **3** or **7** with cyanic bromide respectively. The compound **4** was nitrated with fuming nitric acid (98%) to obtain **5**. Compounds **8a**, **8b** and **8c** were obtained through treatment of **8** in ammonium hydroxide (25%), hydrazine hydrate (80%), and hydroxylamine solution (50%) respectively. All these compounds were fully determined by IR, NMR (^1^H and ^13^C) spectroscopy. The density of these newly synthesized compounds was measured using a gas pycnometer at room temperature. (Yan et al., 2020) The structures of compounds **5**, **8** and **8a** were further determined by single X-ray diffraction.

**FIGURE 1:**
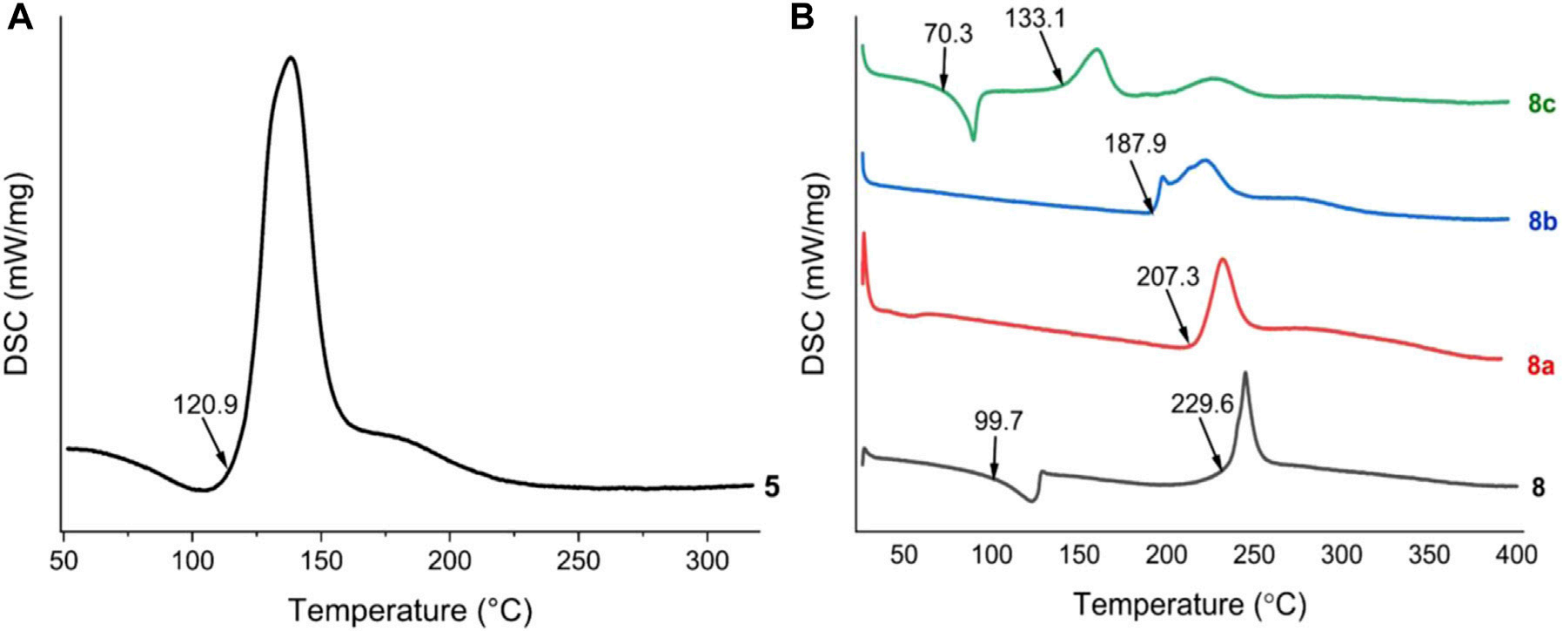
**(A)** DSC plots of compound **5 (B)** DSC plots of compounds of 8, 8a, 8b and 8c (measured at a heating rate of 5°C/min under N_2_ flow).

All these compounds were fully dried at 40°C before testing their physicochemical properties. Differential scanning calorimetry (DSC) was used to determine the thermal stabilities of these energetic materials at a heating rate of 5°C/min under N_2_ flow (Figure 1). **5** exhibits low thermal stabilities (*T*_dec_ = 120.9°C) (Figure 1A). **8** and its energetic salts (**8a** and **8b**) exhibit high thermal stabilities (*T*_dec_ = 187.9–229.6°C), but the decomposition temperature of **8c** is low (133.1°C) (Figure 1B). Compounds **8** and **8c** lost intramolecular crystal water at 99.7°C and 70.3°C, respectively. In general, the storage and working temperatures of munitions are less than 70°C (Yin et al., 2016), so all the compounds can satisfy most military and civilian demands.

Sensitivities (impact and friction) were determined using BAM technology. (Sheng et al., 2015) Compound **5** has high sensitivities (IS = 4.5 J, FS = 59 N), but lower than those of DDNP (IS = 1 J; FS = 24.7 N) and LA (IS = 2.5–4 J; FS = 0.1–1 N) (Table 2), making it more safe in practical application. The heats of formation (HOF) of these compounds were calculated using Gaussian 09 (Revision D.02) program. (Frisch et al., 2009; Fischer et al., 2016) Based on the calculated HOF values and experimental densities, detonation velocities (*D*_v_) and detonation pressures (*P*) were determined using EXPLO5 (v6.01).3. (Suceska, 2013) As shown in Table 2, **5** possess higher density (1.84 g/cm^3^) than DDNP, and better detonation performances (*D*_v_ = 8450 m/s; *p* = 31.6 GPa) than LA and DDNP. Based on these above results, **5** has the potential to become an effective and green primary explosive owing to its safety, good detonation performance and free of heavy metal.

**TABLE 2:** Properties of compound 5.

Compound	Formula	Metal (%)^c^	*ρ* (g/Cm^3^)^d^	*D*_v_ (m/s)^e^	*P* (GPa)^d^	IS (J)^e^	FS(N)^f^	*T*_d_ (°C)^g^
**5**	C_4_H_1_O_3_N_9_	0	1.84	8450	31.6	4.5	59	120.9
**LA** ^h^	N_6_Pb	71.1	4.80	5920	33.8	2.5–4	0.1–1	315
**DDNP** ^i^	C_6_H_2_N_4_O_5_	0	1.72	6900	24.2	1	24.7	157

a metal content.

b density measured at 25°C.

c Calculated detonation velocities (m/s).

d Calculated detonation pressure.

e Impact sensitivity.

f Friction sensitivity.

g decomposition temperature (oneset).

h Lead azide.

i 2-diazo-4, 6-dinitrophenol.

**FIGURE 2:**
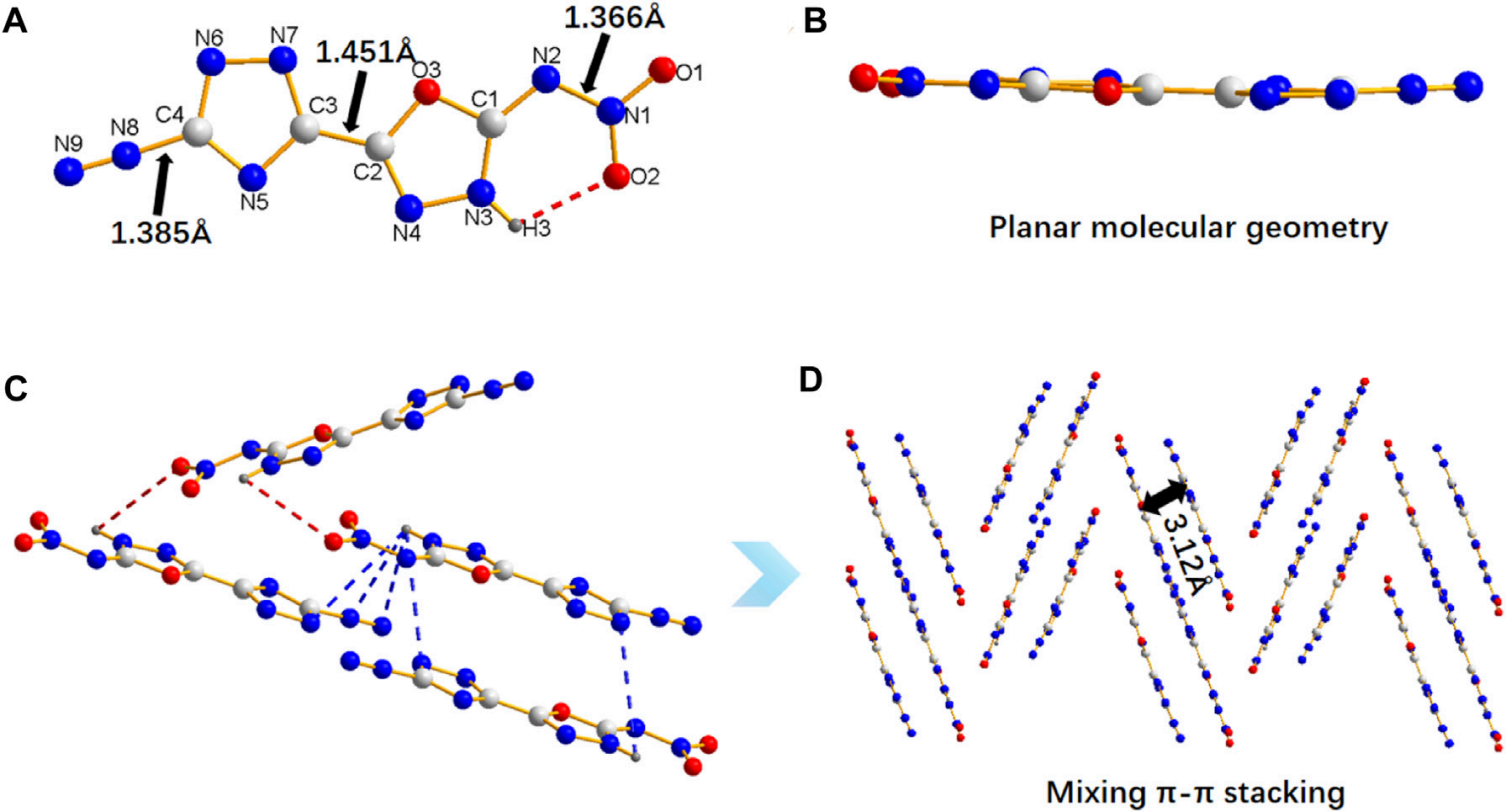
The molecule and crystal stacking structure of 5. **(A)** The crystal structure of 5. **(B)** The planar molecular geometry of 5. **(C)** The intermolecular hydrogen bonds of 5. **(D)** The crystal stacking structure of 5.

The crystal structure of **5** can further explain its properties. The molecular structure of **5** crystallizes in monoclinic system and belong to the *C*2/*c* space group with four molecules per unit cell (*Z* = 4). More details on crystallographic date of **5** are provide in Supplementary Materials (Supplementary Table S1). The compound **5** are nearly coplanar, as confirmed by the torsion angles of 1,2,4-triazole, 1,3,4-oxadiazole (O3-C2-C3-N5: 179.02 (14)°, O3-C2-C3-N7: 0.9 (2)°, N3-C2-C3-N5: 1 (3)°, N4-C2-C3-N7: 177.12 (16)°), and nitro group (O1-N1-N2-C1: 178.09 (15)°, O2-N1-N2-C1: 1.9 (2)°) (Figures 2A,B, Table S2). H3 are bonded to N3 in the oxadiazole ring (Figure 2A). Such structural feature results in intramolecular hydrogen-bonding between H3 and O2 (2.141 Å), which fixes the rotation of nitro group, thereby causing the coplanarity of nitro group with 1,3,4-oxadiazole ring. In addition, there are a large number of intermolecular hydrogen bonds in compound **5** (Figure 2C). The bond of C-N^2+^ (1.385 Å) are slightly longer than common C-N bond (1.321–1.358 Å) and the N-NO_2_ (1.366 Å), and the crystal packing of **5** shows a staggered π-π stacking mode (Figure 2D), both of which are the main reasons for its high sensitivities towards impact and friction. (Zhang et al., 2008)

**FIGURE 3:**
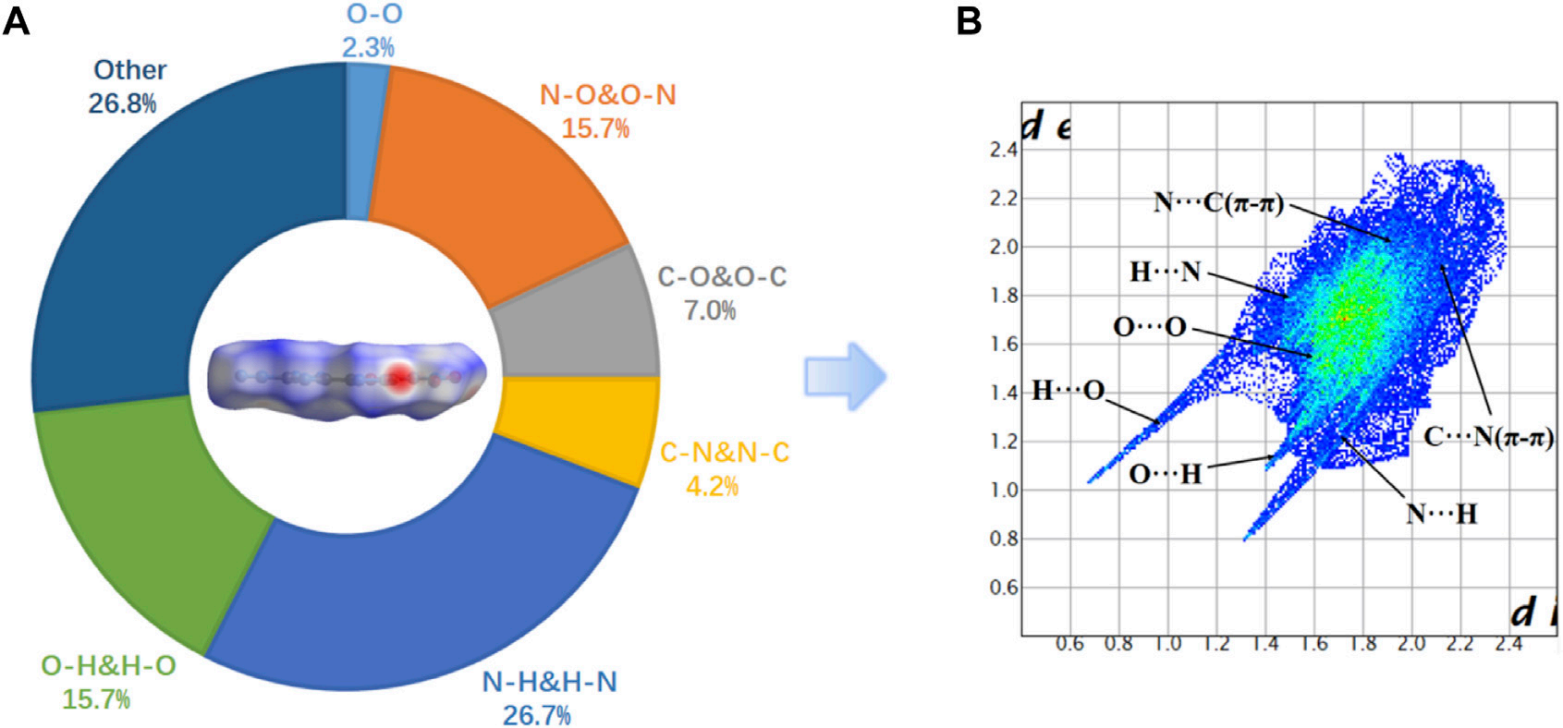
**(A)** Hirshfeld surface and the percentage contribution of every atomic contact for 5; **(B)** fingerprint plot for 5.

The two-dimensional (2D)-fingerprint of crystal **5** and relevant Hirshfeld surfaces were performed to further understand their stability. (Spackman and Jayatilaka, 2009; Tests were conducted according to, 2009; Wang et al., 2018) The 2D fingerprint showed that O ^**…**^ H and N ^**…**^ H hydrogen bonding interactions occupy a smaller proportion 42.4% (Figure 3A).And the C**-**O interaction and C-N interaction (π-π stacking interaction) account for 7% and 4.2% of the total weak interactions, respectively. A higher proportion of the π-π interaction is manifested in its smaller interlayer distance, which results in a higher density of compound 5 (1.84 g/cm^3^). In compound **5**, the N ^**…**^ H and O ^**…**^ H hydrogen bonding interactions occupy a smaller proportion, which is an important reason for the low stability of compound **5**.

Both **8** and **8a** are insensitive (IS > 40 J, FS > 360 N), while **8b** and **8c** have better insensitivity to mechanical stimulation than TNT (**8b**: IS = 23 J; FS = 260 N and **8c:** IS = 19 J; FS = 220 N) (Table 3). Compound **8 and 8c** shows the acceptable detonation velocity and pressure, which are comparable with that of TNT, TATB and RDX. The acceptable detonation performance, insensitivity and thermal stability of **8** demonstrate its potential as new secondary explosives. Further modification of the amino group in **8** can theoretically obtain energetic molecules with better performance. Some attempts have been made by our group, but no desired product has been obtained yet.

**TABLE 3:** Properties of compounds **8** - **8c**.

Compound	*T*_d_ (^o^C)^a^	*ρ* (g/Cm^3^)^b^	Δ*H*_f_(kJ/mol)^c^	*D*_v_ (m/s)^d^	*P* (GPa)^e^	IS(J)^f^	FS (N)^g^
8	229.6	1.78	245	8130	29.2	>40	>360
8a	207.0	1.74	165	7380	23.7	>40	>360
8b	187.9	1.83	289	7962	28.5	23	260
8c	133.1	1.90	219	8320	30.8	19	220
TNT	295	1.65	−59.4	7303	21.3	15	353
RDX	204	1.80	92.6	8795	34.9	7.5	120
TATB	360	1.93	−139.7	8114	30.5	50	>360

a decomposition temperature (onset).

b density measured at 25°C.

c Heat of formation.

d Calculated detonation velocity.

e Calculated detonation pressure.

f Impact sensitivity.

g Friction sensitivity.

**FIGURE 4:**
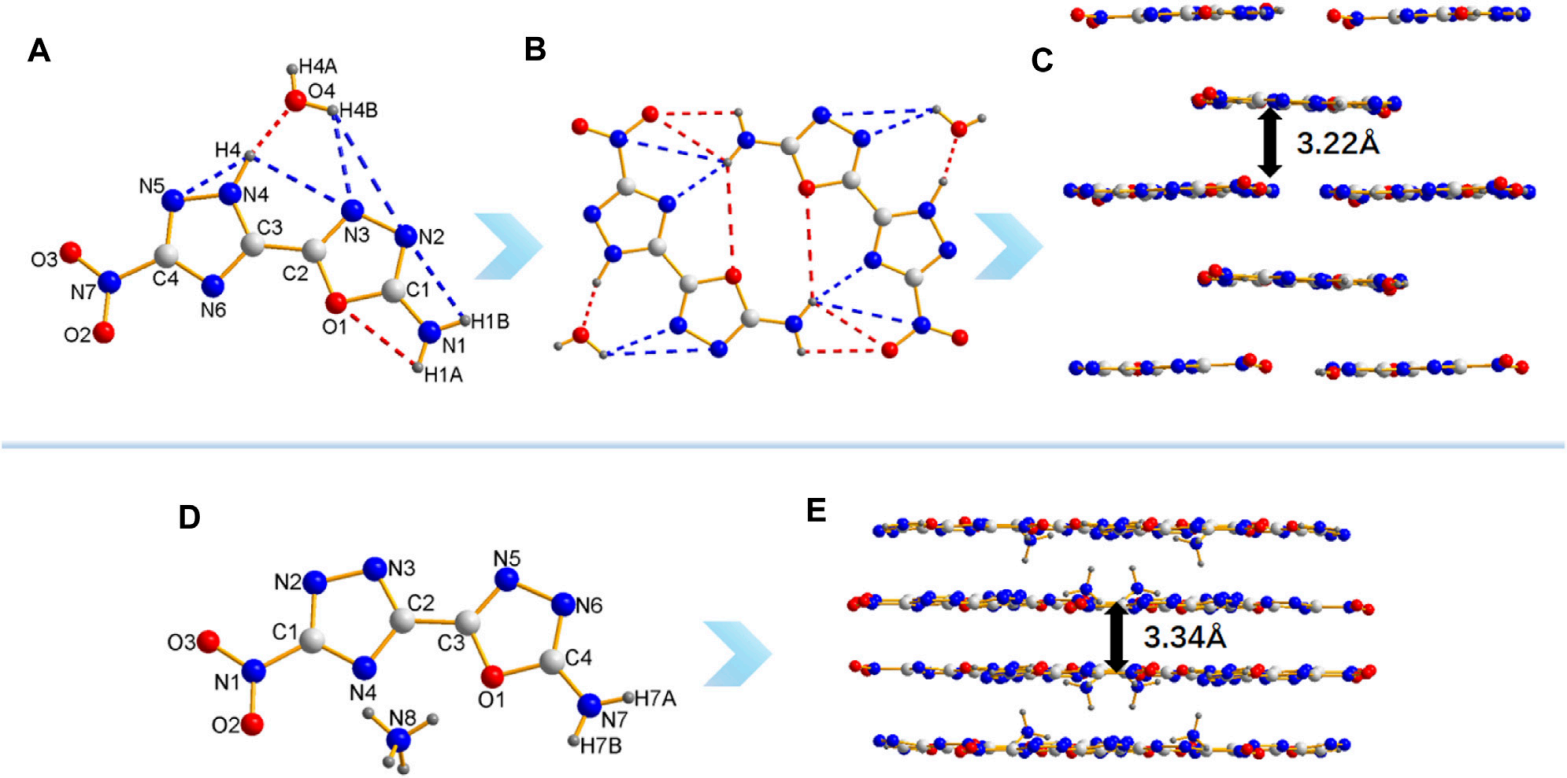
**(A)** The crystal structure and intramolecular hydrogen bonds of 8. **(B)** Intermolecular hydrogen bonds in 8. **(C)** The crystal packing of 8. **(D)** The crystal structure of 8a. **(E)** The crystal packing of 8a.

In order to obtain furtherinsights the relationship between the structure and properties of **8** and **8a**, their single crystal structures were detected by single-crystal X-ray diffraction (Figures 4A,D). The molecules structure of **8** and **8a** crystallize in monoclinic system and belong to the *P*2_1_/*c* and *C*2/*c* space group, with four and eight molecules per unit cell, respectively. More details of crystallographic data are provided in Supplementary Materials (Supplementary Table S1, S3 and S4). The crystal densities of **8** and **8a** are 1.78 g/cm^3^ and 1.74 g/cm^3^, respectively, which are consistent with their theoretical densities. All active hydrogens (H1A, H1B and H4) of **8** and water take part in the formation of intra- and intermolecular hydrogen bonds including N1-H1A ^**…**^ O1, N1-H1B ^**…**^ N2, N4-H4 ^**…**^ N3, N4-H4 ^**…**^ N5, N4-H4···O4, O4-H4B ^**…**^ N3 O4-H4B ^**…**^ N2 (Figures 4A,B). The intermolecular hydrogen bonding interactions are beneficial to increase stability of molecules. The molecular structure of both **8** and **8a** are nearly coplanar, and conjugated systems which are verified by the torsion angles of O2-N7-C4-N5 -5.8 (4)°, N3-C2-C3-N4 2.2 (4)°; C2-O1-C1-N1 -179.4 (2)° of compound **8** (Table S3), the compound **8a** can be verified by O2-N1-C1-N2 -177.48 (12)°; N3-C2-C3-N5 2.3 (2)°; N4-C2-C3-N1 -1.74.44 (13)°; N5-N6-C4-N7 -179.86 (14)° (Table S4). As for the packing diagram (Figures 4C,E), both **8** and **8a** have face-to-face stacking and the distance between two planes are 3.22 Å and 3.34 Å respectively. The geometrical distance of traditional aromatic *π-π* interactions is 3.65–4.00 Å (Yan et al., 2020), so there are strong π-π interactions between layers, which may explain their high crystal density and insensitivity to mechanical stimulation to a certain extent.

**FIGURE 5:**
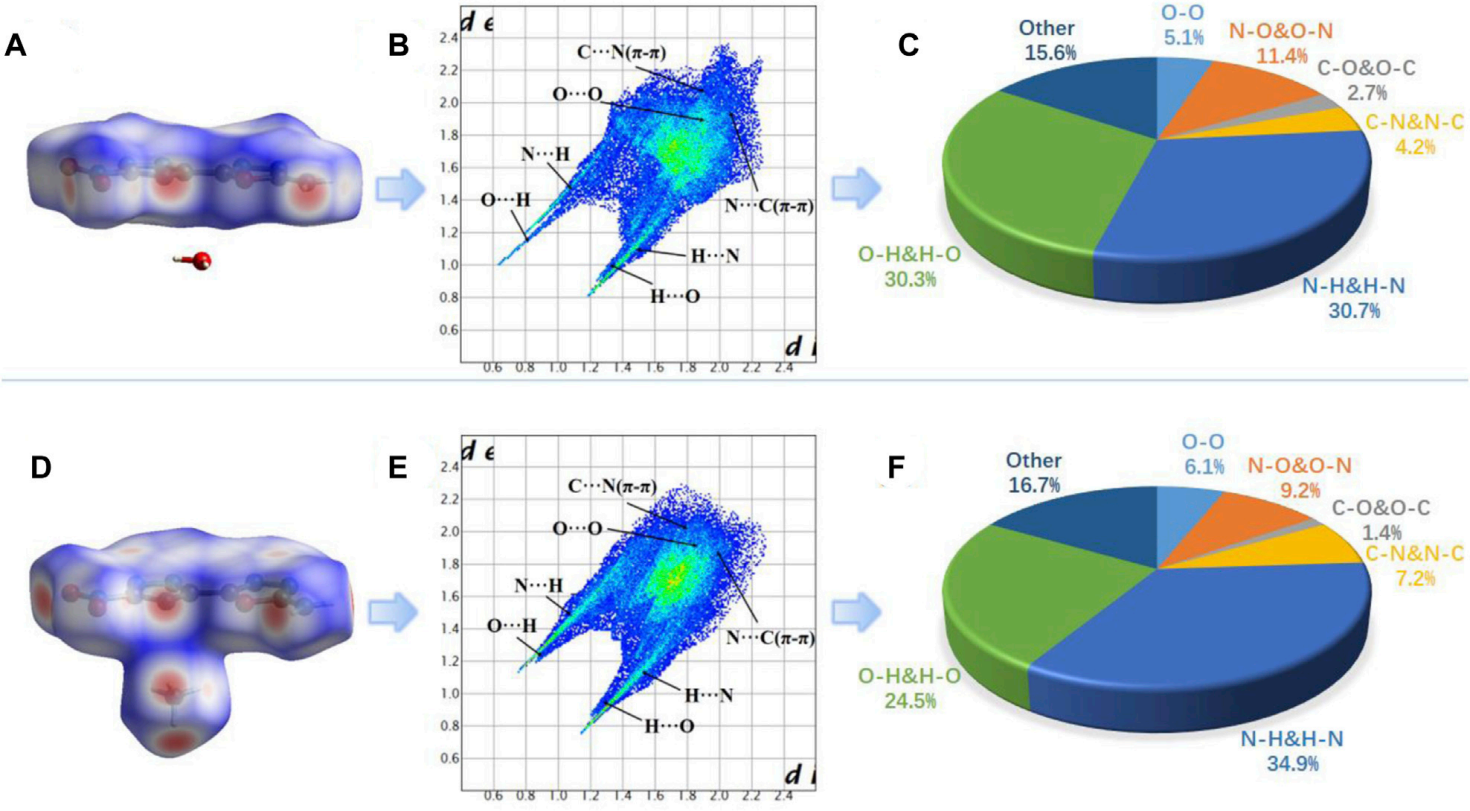
**(A)**, **(D)** Hirshfeld surface of 8 and 8a; **(B), (E)** fingerprint plot for 8 and 8a; **(C), (F)** the percentage contribution of every atomic contact for 8 and 8a.

Similarly, the two-dimensional (2D)-fingerprint of crystals **8** and **8a** and relevant Hirshfeld surfaces also were used to further understand their stability. Both **8** and **8a** contain planar π-conjugated structure and appear in plate shapes, which is helpful for enhancing π-π stacking (Zhang et al., 2013) (Figures 5A,D). It is observed from the 2D fingerprint plot that O ^**…**^ H and N ^**…**^ H hydrogen bonding interactions (a pair of remarkable spikes on the bottom left) constitute 61.0% (**8**) and 59.4% (8a) of the total weak interactions, respectively (Figures 5B,E). The C-O interaction (π-π stacking interaction) ratio of 8 (2.7%) is higher than that of **8a** (1.4%). The ratio of O-O interactions in 8a is 6.1%, which is higher than that in 8 (5.1%). There are two conclusions according to the above results. (1) High ratio of hydrogen bonding interactions and strong π-π stacking interactions contribute greatly to the molecular stability of **8** and **8a**. (2) **8** contains higher hydrogen bonding and C-O interactions but lower O-O interactions than 8a, so **8** should be more stable than **8a** (Zhang et al., 2015), which is consistent with experimental results.

## 3 Synthesis

Caution: All the products are dangerous materials, explosions of which may occur in certain conditions. Although we have no difficulties on synthesizing and handing the compounds, proper safety precautions such as safety glasses, plastic spatulas and face shields must be used, especially when the experiments is performed on a large scale.

Synthesis of 5-(5-amino-1H-1,2,4-trizol-3-yl)-1,3,4-oxadiazol-2-amine (4): Compound 3 (2.84 g 20 mmol) and potassium bicarbonate (2.21g, 22 mmol ) were slurried with methanol (30 ml) at room temperature then cyanogen bromide (2.12 g, 20 mmol) was added dropwise. It was stirred at room temperature for 72 h, then the temperature was raised to reflux for another 72 h. After reaction completed, the resulting yellow precipitate was filtered, washed with water and dried to yield **4** (2.84 g, 85%). ^1^H NMR (500 MHz DMSO-d_6_): δ = 6.26 (s, 2H NH_2_) 7.21 (s, 2H NH_2_) and 12.50 (s, 1H, NH) ppm. ^13^C NMR (126 MHz DMSO-d_6_): δ = 148.00, 152.81, 157.94 and 164.02 ppm. IR (KBr): ṽ 3680, 3320, 3030, 2930, 1650, 1540, 1280, 1020, 835, 768, 577, 549, 474, 468, 430, 416, 409 cm^−1^.

Synthesis of 5-(diazo-1,2,4-triazol-3-yl)-1,3,4-oxadiazol-2-nitroamino (5): Compound 4 (1g 6 mmol) was added by portions into smoothly stirred fuming HNO_3_ (98% 8 ml) which was cooled by ice. Then, the reaction was allowed to warm to room temperature and continued for about 48 h. After a yellow precipitate appeared, the mixture was poured into ice water and filtered to obtain a yellow precipitate. After washing with small amount of water, the yellow solid was dried naturally, and pure 5-(diazo 1,2, 4-triazol-3-yl)-1,3,4-oxadiazol-2-nitroamino was obtained **5** as a yellow powder (1.1 g, 82%). ^13^C NMR (126 MHz DMSO-d_6_): δ = 135.79, 150.56, 150.83 and 161.82 ppm. DSC (5°C/min): T_dec._ = 120.9°C. IR (KBr): ṽ 3470, 3400, 3340, 2990, 2930, 2250, 1670, 1580, 1470, 1350, 1310, 1200, 1090, 1020, 996, 946, 853, 797, 755, 734, 637, 578, 532, 446, 421 cm^−1^

Synthesis of ethyl-nitro-1H-1,2,4-triazole-3-carboxylate (6): Compound 2 (0.5 g, 30 mmol) in 20% sulfuric acid (6 ml) was added dropwise to a solution of sodium nitrite (30 equiv, 6.8 g, 98 mmol) in water (10 ml) at 40°C. The mixture was stirred at 50°C for 1 h. After cooling down to room temperature, the mixture was acidified with sulfuric acid (20%) until no evolution of NO_2_ could be observed. The reaction mixture was extracted with ethyl acetate, dried over MgSO_4_, and the solvent was evaporated to yield **6** (0.41 g, 74%) as a yellow solid. ^1^H NMR (500 MHz DMSO-d_6_): δ = 1.32 (s, 3H, CH_3_), 4.39 (s, 2H, CH_2_) ppm. ^13^C NMR (126 MHz DMSO-d_6_): δ = 14.29, 63.09, 148.03, 156.51 and 162.63 ppm.

Synthesis of sodium 5-nitro-1,2,4-triazole-3-carbohydrazide (7): Compound 6 (10 g, 54 mmol) was added to a 100 ml saturated sodium hydrogen carbonate solution, and the solvent was evaporated. Ethyl alcohol 50 ml was added and stirring 0.5h, The insoluble matter was removed by filtration, then hydrazine hydrate (3 equiv, 9.5 g, 162 mmol) was added the temperature was raised to reflux maintain 2h. Cooled to room temperature and the precipitated solid was filtered, air-dried to afford **7** as a yellow solid (9.2 g, 88%). ^1^H NMR (500 MHz DMSO-d_6_): δ = 4.42(s, 2H, NH_2_), 9.47(s, 1H, NH) ppm. ^13^C NMR (126 MHz DMSO-d_6_): δ = 157.06, 160.87 and 165.66 ppm.

Synthesis of 5-(5-nitro -1H-1,2,4-trizol-3-yl)-1,3,4-oxadiazol-2-amine (8): Compound 7 (3.88 g, 20 mmol) potassium bicarbonate (2.21 g, 22 mmol ) was slurried with methyl alcohol (30 ml) at room temperature. Then cyanogen bromide (2.12 g, 20 mmol) was added dropwise. It was stirred at room temperature for 72 h, then the temperature was raised to reflux for another 72 h. The concentrated hydrochloric acid (1.97 g, 20 mmol) was added stirring another 0.5h at room temperature, the resulting yellow precipitate was filtered, washed with water and dried to yield **8** (2.84 g, 72%). ^1^H NMR (500 MHz DMSO-d_6_): δ = 7.76 (s, 2H, NH_2_) ppm. ^13^C NMR (126 MHz DMSO-d_6_): δ = 144.57, 148.91, 163.16 and 165.02 ppm. DSC (5°C/min): T_dec._ = 229.6°C. IR (KBr): ṽ 3670, 3330, 3160, 2990, 2900, 1670, 1620, 1500, 1410, 1380, 1310, 1060, 1030, 1010, 950, 844, 706, 647, 533, 490, 472, 436, 430 cm^−1^

General procedure for synthesis of salts (8a-8c)

To a water/methanol solution (7 ml/7 ml) of **8** (300 mg, 1.52 mmol), the corresponding base (25% ammonia solution (0.21 g, 1.52 mmol), 85% hydrazine hydrate (0.09 g, 1.52 mmol), 50% hydroxylamine solution (0.1 g, 1.52 mmol)) was added. The resulting solution was stirred at room temperature for 30 min, then the solids were collected by filtration, dried at room temperature.

Ammonia 3-(5-amino-1,3,4-oxadiazol-2-yl)-5-nitro-1,2,4-triazolate hydrate (8a)

Yield 80% (260 mg, 1.22 mmol) as a yellow solid.

^1^H NMR (500 MHz DMSO-d_6_): δ = 7.27 (s, 2H, NH_2_). ^13^C NMR (126 MHz DMSO-d_6_): δ = 150.79, 153.66, 163.85 and 166.17 ppm. DSC (5°C/min): T_dec._ = 207.0°C.

Hydrazine 3-(5-amino-1,3,4-oxadiazol-2-yl)-5-nitro-1,2,4-triazolate hydrate (8b)

Yield 75% (260 mg, 1.14 mmol) as a yellow solid.

^1^H NMR (500 MHz DMSO-d_6_): δ = 7.27 (s, 2H, NH_2_). ^13^C NMR (126 MHz DMSO-d_6_): δ = 150.79, 153.66, 163.85 and 166.17 ppm. DSC (5°C/min): T_dec._ = 187.9°C.

Hydroxylamine 3-(5-amino-1,3,4-oxadiazol-2-yl)-5-nitro-1,2,4-triazolate hydrate (8c)

Yield 81% (283 mg, 1.23 mmol) as a yellow solid.

^1^H NMR (500 MHz DMSO-d_6_): δ = 7.27 (s, 2H, NH_2_). ^13^C NMR (126 MHz DMSO-d_6_): δ = 150.79, 153.66, 163.85 and 166.17 ppm. DSC (5°C/min): T_dec._ = 133.1°C.

## 4 Conclusion

A novel family of energetic derivatives based on 2-(1,2,4-triazol-5-yl)-1,3,4-oxadiazole as the building block have been developed. Compound **5** possesses a low decomposition temperature (*T*_dec_ = 120.9°C) and high mechanical sensitivity (IS = 4.5 J; FS = 59 N), and has several advantages over conventional primary explosives including good detonation performance and free of heavy metal, so it has potential prospects in the application of primary explosives. The presence of diazo group and staggered *π-π* stacking mode are the main reasons for its high sensitivities towards impact and friction. The good thermal stability and insensitivity of **8** and **8a** is owing to the presence of extensive hydrogen-bonding and strong *π–π* stacking interactions. Although the detonation performance of **5** and **8** is lower than that of RDX, their structure can be further improved. For example, the diazo group of **5** can be further cyclized with nitroacetonitrile or malononitrile and its amino group of **8** can be nitrated, which may further enhance the detonation performance of this molecule. Follow-up work is going on in our group.

### Accession codes

CCDC 2180404, 2180405 and 2180395 contain the supplementary crystallographic date for this paper. There date can be obtained free of charge via https://www.ccdc.cam.ac.uk/structures/.

## Data Availability

The datasets presented in this study can be found in online repositories. The names of the repository/repositories and accession number(s) can be found in the article/Supplementary Material.
