# Supplementary material for: 2-(1,2,4-triazole-5-yl)-1,3,4-oxadiazole as a novel building block for energetic materials
Source: Front Chem. 2022 Aug 26;10:996812. doi: 10.3389/fchem.2022.996812 (PMC9458958; doi:10.3389/fchem.2022.996812)
Supplement: Supplementary file 1 [file DataSheet1.docx]

Supplementary Material

2-(1,2,4-triazole-5-yl)-1,3,4-oxadiazole as a novel building block for energetic materials

Zheting Dong,^a^ Zhengqiang Wu,^a^ Qiang Zhang,^b^ Yuangang Xu,^a^* Guo-Ping Lu^a^*

^a^ School of Chemistry and Chemical Engineering, Nanjing University of Science and Technology, Nanjing 210094, China

^b^ School of Chemistry and Life Sciences, Suzhou University of Science and Technology, Suzhou 215009, China

*** Correspondence:**Corresponding Author
[glu@njust.edu.cn](mailto:glu@njust.edu.cn); [yuangangxu@163.com](mailto:yuangangxu@163.com)

# 1 Experimental Section

All reagents and solvents were purchased from Aladdin and Energy Chemical as analytical grade and were used as received. ^1^H and ^13^C NMR spectra were recorded on a Bruker 500 MHz (Bruker AVANCE 500) nuclear magnetic resonance spectrometer. Chemical shifts for ^1^H and ^13^C NMR are reported relative to Me4Si as external standards. The melting and decomposition (onset) points were obtained at a heating rate of 5 ℃/ min on a differential scanning calorimeter (Mettler Toledo DSC823e) in closed Al containers with a nitrogen flow of 50 ml/min . IR spectra were recorded using KBr pellets for solids on a Thermo Nicolet iS10 spectrometer. Elemental analyses were carried out on a vario EL ℃ CHNOS elemental analyzer. Impact and friction sensitivity measurements were made using a standard BAM fallhammer and a BAM friction tester.

# 2 X-ray crystallography detail

A yellow crystal **(5)** of dimensions 0.15x0.13x0.12 mm^3^, a yellow crystal **(8)** of dimensions 0.13x0.11x0.1 mm^3^, a yellow crystal **(8a)** of dimensions 0.16x0.15x0.12 mm^3^ were collected with a Bruker D8 VENTURE diffractometer with graphite-monochromated Mo-Ka radiation (λ=0.71073 nm). The crystal was kept at 193.00 K during data collection. Using Olex2^[1]^, the structure was solved with the SHELXT structure solution program using Intrinsic Phasing and refined with the SHELXL^[2]^ refinement package using Least Squares minimisation.

**Table S1**. Crystallographic data.

| Compound | **5** | **8** | **8a** |
| --- | --- | --- | --- |
| CCDC number  formula  Mw  crystal system  space group  a [Å]  b [Å]  c [Å]  α [°]  β [°]  γ [°]  V [Å^3^]  Z  T [K]  λ [Å]  ρ_calcd_ [g/cm^3^]  μ [mm^-1^]  F [000]  2θ range [°]  GOF on F^2^  R_1_[I>2σ(I)]  WR_2_[I>2σ(I)]  R_2_[all data]  WR_2_[all data] | 2180405  C11H14N18O9  542.40  monoclinic  C2/c  15.621(8)  8.480(4)  16.740(9)  90  90.80(2)  90  2217.1(19)  4  193  0.71073  1.625  0.141  1112.0  4.868-55.226  1.087  0.0499  0.1283  0.0677  0.1398 | 2180404  C4H5N7O4  215.15  monoclinic  P2_1_/c  11.2392(9)  9.2472(5)  8.1647(6)  90  108.670(30)  90  803.91(10)  4  193  0.71073  1.778  0.157  440.0  3.826-55.078  1.048  0.0454  0.0983  0.0756  0.1225 | 2180395  C4H6N8O3  214.17  Monoclinic  C2/c  12.5472(5)  9.1307(3)  14.5848(6)  90  102.393(2)  90  1631.97(11)  8  193  0.71073  1.743  0.149  880.0  5.564-54.994  1.111  0.0349  0.0830  0.0419  0.0899 |

**Table S2**. Bond lengths [Å] and angles [°] of **5**

| O1-N1 | 1.229(19) | C1-O3-C2 | 104.56(13) |
| --- | --- | --- | --- |
| O2-N1 | 1.237(2) | O1-N1-O2 | 123.20(16) |
| O3-C1 | 1.354(2) | O1-N1-N2 | 115.07(15) |
| O3-C2 | 1.370(2) | O2-N1-N2 | 121.73(14) |
| N1-N2 | 1.366(2) | C1-N2-N1 | 114.78(14) |
| N2-C1 | 1.321(2) | C1-N3-N4 | 110.62(13) |
| N3-N4 | 1.388(19) | C2-N4-N3 | 103.55(14) |
| N3-C1 | 1.325(2) | C4-N5-C3 | 97.20(15) |
| N4-C2 | 1.282(2) | C4-N6-N7 | 102.78(13) |
| N5-C3 | 1.339(2) | N6-N7-C3 | 105.80(14) |
| N5-C4 | 1.335(2) | N9-N8-C4 | 177.43(18) |
| N6-N7 | 1.341(2) | N2-C1-O3 | 114.78(15) |
| N6-C4 | 1.338(2) | N2-C1-N3 | 137.68(16) |
| N7-C3 | 1.358(2) | N3-C1-O3 | 107.50(14) |
| N8-N9 | 1.093(2) | O3-C2-C3 | 117.25(14) |
| C2-C3 | 1.451(2) | N4-C2-O3 | 113.76(15) |
| C3-C4 | 2.006(2) | N4-C2-C3 | 128.97(15) |
| C6-O5 | 1.351(3) | N5-C3-N7 | 115.47(15) |
| O4-C5 | 1.417(3) | N5-C3-C2 | 123.41(15) |
|  |  | N5-C3-C4 | 41.32(9) |
|  |  | N7-C3-C2 | 121.10(15) |
|  |  | N7-C3-C4 | 74.14(11) |
|  |  | C2-C3-C4 | 164.67(14) |
|  |  | N5-C4-N6 | 118.76(15) |
|  |  | N5-C4-N8 | 121.87(16) |
|  |  | N5-C4-C3 | 41.47(9) |
|  |  | N6-C4-N8 | 119.36(15) |
|  |  | N6-C4-C3 | 77.28(11) |
|  |  | N8-C4-C3 | 163.31(14) |

**Table S3**. Bond lengths [Å] and angles [°] of **8**

| O1-C1 | 1.362(3) | C1-O1-C2 | 101.63(16) |
| --- | --- | --- | --- |
| O1-C2 | 1.365(3) | C1-N2-N3 | 105.64(17) |
| O2-N7 | 1.222(2) | C2-N3-N2 | 105.97(18) |
| O3-N7 | 1.219(3) | C3-N4-N5 | 109.45(17) |
| N1-C1 | 1.323(3) | C4-N5-N4 | 101.09(17) |
| N2-N3 | 1.405(3) | C3-N6-C4 | 100.11(18) |
| N2-C1 | 1.316(3) | O2-N7-C4 | 117.69(19) |
| N3-C2 | 1.285(3) | O3-N7-O2 | 124.73(19) |
| N4-N5 | 1.348(2) | O3-N7-C4 | 117.57(18) |
| N4-C3 | 1.339(3) | N1-C1-O1 | 119.1(2) |
| N5-C4 | 1.313(3) | N2-C1-O1 | 112.72(18) |
| N6-C3 | 1.329(3) | N2-C1-N1 | 128.2(2) |
| N6-C4 | 1.341(3) | O1-C2-C3 | 120.45(18) |
| N7-C4 | 1.452(3) | N3-C2-O1 | 114.03(19) |
| C2-C3 | 1.455(3) | N3-C2-C3 | 125.5(2) |
|  |  | N4-C3-C2 | 121.94(19) |
|  |  | N6-C3-N4 | 111.41(18) |
|  |  | N6-C3-C2 | 126.6(2) |
|  |  | N5-C4-N6 | 117.94(19) |
|  |  | N5-C4-N7 | 119.58(19) |
|  |  | N6-C4-N7 | 122.48(19) |

**Table S4**. Bond lengths [Å] and angles [°] of **8a**

| O1-C3 | 1.3648(15) | C4-O1-C3 | 102.42(10) |
| --- | --- | --- | --- |
| O1-C4 | 1.3614(15) | O2-N1-O3 | 123.91(12) |
| O2-N1 | 1.2246(15) | O2-N1-C1 | 118.80(12) |
| O3-N1 | 1.2287(15) | O3-N1-C1 | 117.29(12) |
| N1-C1 | 1.4395(17) | C1-N2-N3 | 104.13(10) |
| N2-N3 | 1.3526(16) | C2-N3-N2 | 105.52(11) |
| N2-C1 | 1.3242(17) | C1-N4-C2 | 98.64(11) |
| N3-C2 | 1.3426(17) | C3-N5-N6 | 106.46(11) |
| N4-C1 | 1.3354(16) | C4-N6-N5 | 105.49(11) |
| N4-C2 | 1.3481(17) | N1-C1-C2 | 163.64(11) |
| N5-N6 | 1.4111(15) | N2-C1-N1 | 120.13(12) |
| N5-C3 | 1.2837(17) | N2-C1-N4 | 117.05(12) |
| N6-C4 | 1.3096(17) | N2-C1-C2 | 76.14(8) |
| N7-C4 | 1.3230(18) | N4-C1-N1 | 122.80(12) |
| C1-C2 | 2.0351(18) | N4-C1-C2 | 40.91(7) |
| C2-C3 | 1.4525(18) | N3-C2-N4 | 114.66(12) |
|  |  | N3-C2-C1 | 74.21(8) |
|  |  | N3-C2-C3 | 121.76(12) |
|  |  | N4-C2-C1 | 40.44(7) |
|  |  | N4-C2-C3 | 123.51(12) |
|  |  | C3-C2-C1 | 163.75(11) |
|  |  | O1-C3-C2 | 118.48(11) |
|  |  | N5-C3-O1 | 113.10(11) |
|  |  | N5-C3-C2 | 128.39(12) |
|  |  | N6-C4-N7 | 112.52(11) |
|  |  | N6-C4-N7 | 129.97(13) |
|  |  | N7-C4-O1 | 117.52(12) |

# 3 Theoretical study

All of the ab initio calculations involved in this work were carried out using the Gaussian 09 suite of programs.^[3]^ The geometric optimization and frequency analysis of the structures are based on available single-crystal structures and using the B3LYP functional with the 6-311++G (d, p) basis set. The geometrical were optimized with no constraints imposed under default convergence criteria. Total energy (E0) and zero-point energy (ZPE) were calculated with vibrational frequency analysis. Atomization energies were obtained by employing the G2 ab initio method. All of the optimized structures were characterized to be true local energy minima on the potential energy surface without imaginary frequencies. For energetic salts, the solid-phase heat of formation is calculated based on a Born-Haber energy cycle (**Scheme S1**) [2]. The number is simplified by equation 1:

**Scheme S1**. Born-Haber Cycle for the formation of energetic salts.

**∆Hfº (salt, 298 K) = ∆Hfº (cation, 298K) + ∆H_f_º (anion, 298K) – ∆H_L_ (1)**

where ∆H_L_ is the lattice energy of the salts, which could be predicted by using the formula suggested by Jenkins et al.^[4]^ [Eq. (2)]

**∆H_L_ = U_POT_ + [p(n_M_/2 – 2) + q(n_X_/2 – 2)]RT (2)**

where n_M_ and n_X_ depend on the nature of the ions, Mq^+^ and Xp^-^ , respectively. The equation for lattice potential energy UPOT [Eq. (3)] has the form:

**U_POT_ [kJ mol^-1^ ] = γ(ρ_m_/M_m_)^1/3^ + δ (3)**

where ρ_m_ [g cm^−3^ ] is the density of the salt, Mm is the chemical formula mass of the ionic material, and values for (g) and the coefficients γ (kJ mol^−1^ cm) and δ (kJ mol^−1^) are assigned literature values.

The solid-state enthalpy of formation for neutral compound can be estimated by subtracting the heat of sublimation from gas-phase heat of formation. Based on the literature^[5,6]^, the heat of sublimation can be estimated with Trouton’s rule according to supplementary equation 1, where T represents either the melting point or the decomposition temperature when no melting occurs prior to decomposition:

**ΔH_sub_ = 188/J mol^-1^ K^-1^ × T**

**4 NMR and IR spertra**

^1^H NMR spectra in DMSO-d_6_ for **4**.

^13^C NMR spectra in DMSO-d_6_ for **4**.

^13^C NMR spectra in DMSO-d_6_ for **5**.

^1^H NMR spectra in DMSO-d_6_ for **8**.

^13^C NMR spectra in DMSO-d_6_ for **8**.


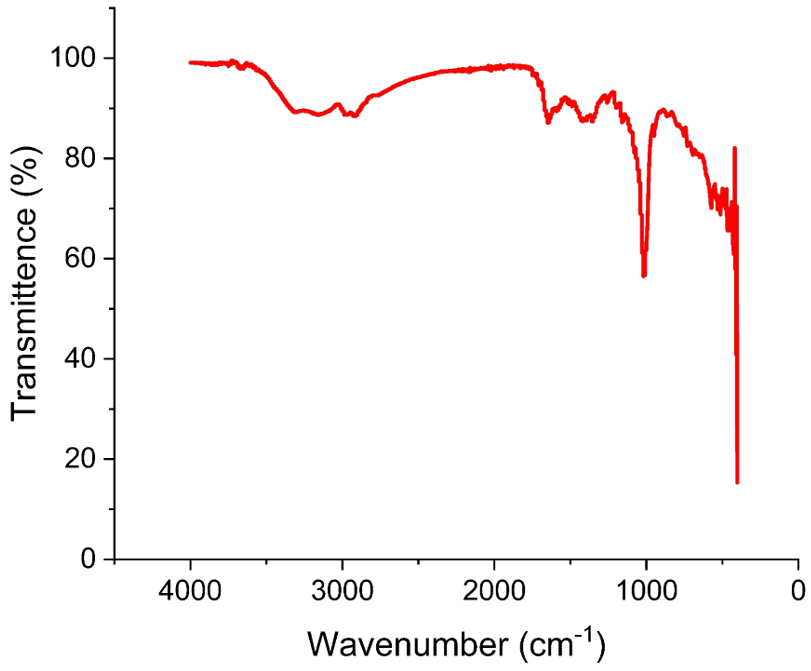


IR Spectra of **4**.


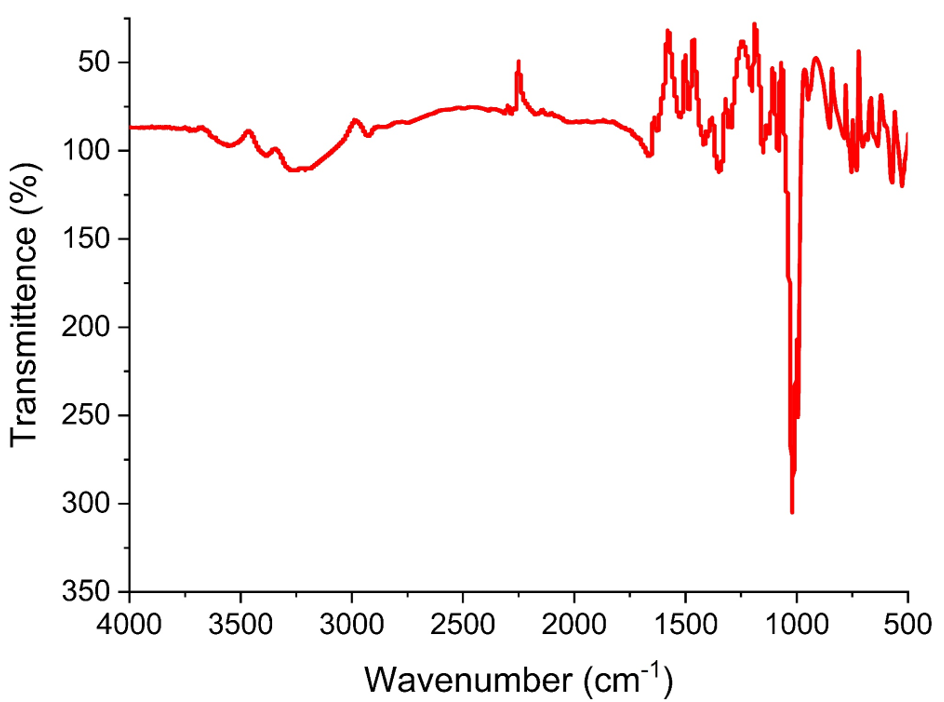


IR Spectra of **5**


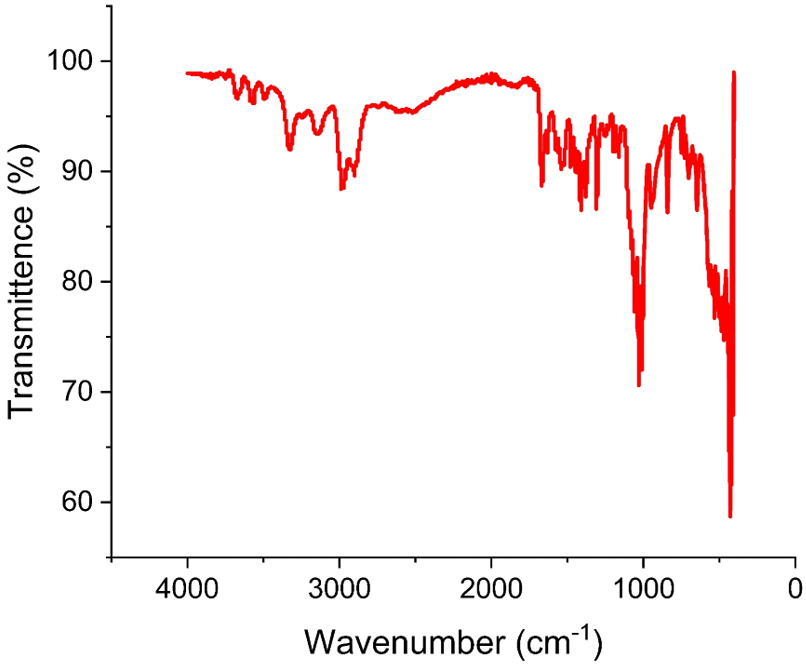


IR Spectra of **8**

# References

1. Dolomanov O V, Bourhis L J, Gildea R J, et al. *OLEX2: a complete structure solution, refinement and analysis program[J]. Journal of applied crystallography,* 2009, 42(2): 339-341. Doi: [10.1107/S0021889808042726](https://doi.org/10.1107/S0021889808042726).
2. Sheldrick G M. *Crystal structure refinement with SHELXL[J]. Acta Crystallographica Section C: Structural Chemistry,* 2015, 71(1): 3-8. Doi: [10.1107/S2053229614024218](http://dx.doi.org/10.1107/S2053229614024218).
3. Frisch MJ, Trucks GW, Schlegel HB, et al. *Gaussian 09, Revision A.02, Gaussian, Inc., Wallingford CT, 2009.*
4. Jenkins H D B, Tudela D, Glasser L. *Lattice potential energy estimation for complex ionic salts from density measurements[J]. Inorganic Chemistry,* 2002, 41(9): 2364-2367. Doi: [10.1021/ic011216k](https://doi.org/10.1021/ic011216k).
5. Trouton F. IV. *On molecular latent heat[J]. The London, Edinburgh, and Dublin Philosophical Magazine and Journal of Science,* 1884, 18(110): 54-57.
6. Westwell M S, Searle M S, Wales D J, et al. Empirical correlations between thermodynamic properties and intermolecular forces[J]. Journal of the American Chemical Society, 1995, 117(18): 5013-5015. Doi: 10.1021/ja00123a001.
